# Supplementary material for: An iris diaphragm mechanism to gate a cyclic nucleotide-gated ion channel
Source: Nat Commun. 2018 Sep 28;9:3978. doi: 10.1038/s41467-018-06414-8 (PMC6162275; doi:10.1038/s41467-018-06414-8)
Supplement: Supplementary file 1 — Supplementary Information [file 41467_2018_6414_MOESM1_ESM.pdf]

Supplementary Information

An iris diaphragm mechanism to gate a cyclic nucleotide-gated ion channel

Arin Marchesi[1], Xiaolong Gao[2] ,Ricardo Adaixo[3], Jan Rheinberger[2]
Henning Stahlberg[5], Crina Nimigean[2,3,4,*] and Simon Scheuring[1,2,3,*]

[1] INSERM U1006, Aix-Marseille Université, Parc Scientifique et Technologique de Luminy, 163 Avenue de Luminy, 13009 Marseille, France.
[2] Department of Anesthesiology, Weill Cornell Medical College, 1300 York Ave, New York, NY 10065, USA.
[3] Center for Cellular Imaging and NanoAnalytics, Biozentrum, University of Basel, Mattenstrasse 26, CH-4058 Basel, Switzerland.
[4]Department of Physiology and Biophysics, Weill Cornell Medical College, 1300 York Ave, New York, NY 10065, USA.
[5] Department of Biochemistry, Weill Cornell Medical College, 1300 York Ave, New York, NY 10065, USA.

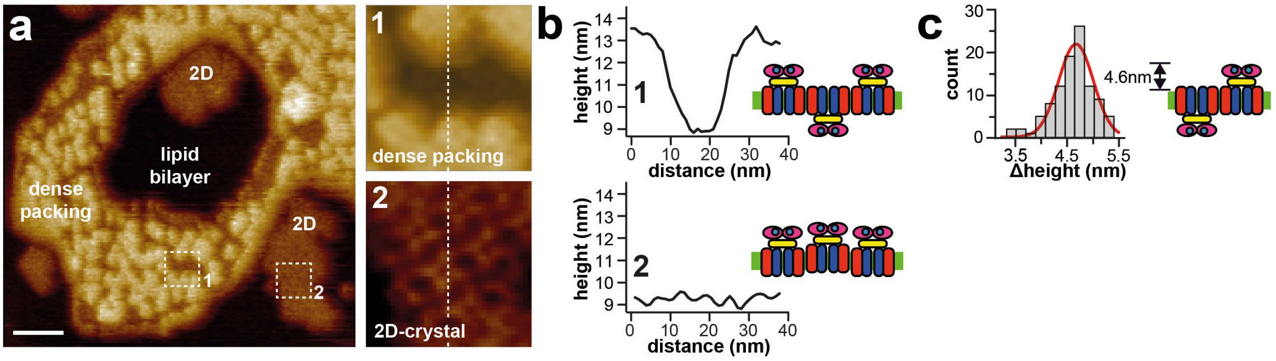

Supplementary Figure 1. Representative overview topograph of membrane-reconstituted SthK channels. (a) HS-AFM image showing the coexistence within the sample of bare lipid bilayer patches, membrane areas densely packed with up- and down-oriented SthK channels (see inset 1) and membrane areas in which the SthK channels are crystallized in 2D-lattices (see inset 2). The small crystals represented approximatively 10% of the observed sample (with large variations between different preps), with the other 90% displaying the alternating up-and-down packing. Imaging buffer: 100mM KCl, 20mM HEPES (pH 8.0), 0.1mM cAMP. Scale bar: 50nm. **(b)** Height profile analysis along the white dashed lines in insets 1 and 2. The densely packed regions with up- and down-packed channels display strong surface corrugation of ~5nm amplitude due to the presence of CNBDs protruding to both sides of the membrane, while the 2D-crystal displays a rather flat overall surface corrugation (see sketches on the right of the height profiles). **(c)** Height difference distribution of up- and down-reconstituted channels within the densely packed areas. The histogram was fitted with a Gaussian function resulting in 4.6±0.5nm height of the CNBD domain above membrane (see sketch on the right of the histogram).

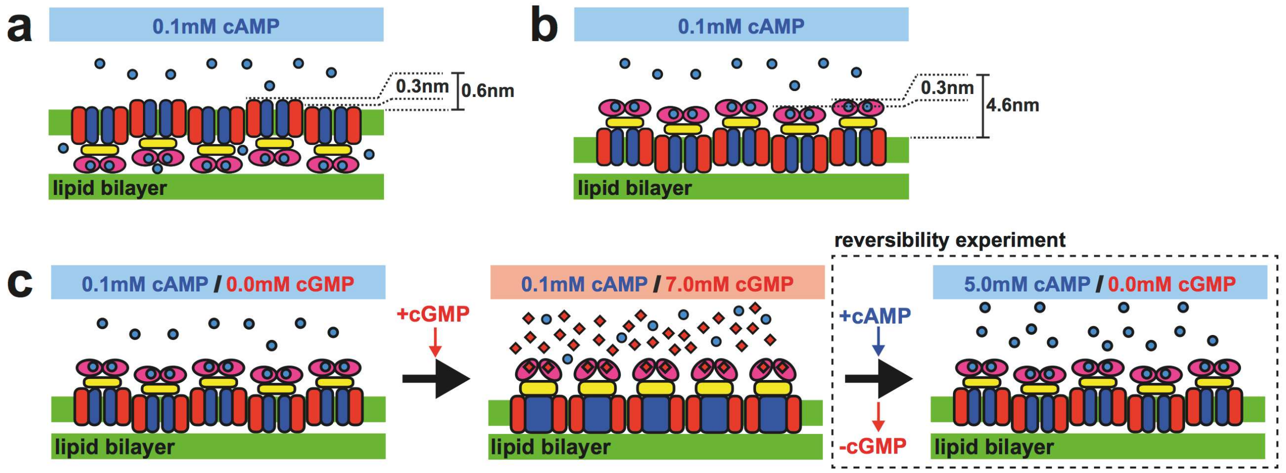

Supplementary Figure 2. 2D-crystal packing cartoons of SthK. SthK packing viewed in the membrane plane (color scheme as in figure 1) with the channels exposing either the extracellular face **(a)** or the CNBD domains **(b)** to the AFM tip. (**c**) Molecular rearrangements reflected in the crystal packing viewed in the membrane plane upon cGMP addition (middle panel), and subsequent cGMP replacement with saturating cAMP (boxed area).

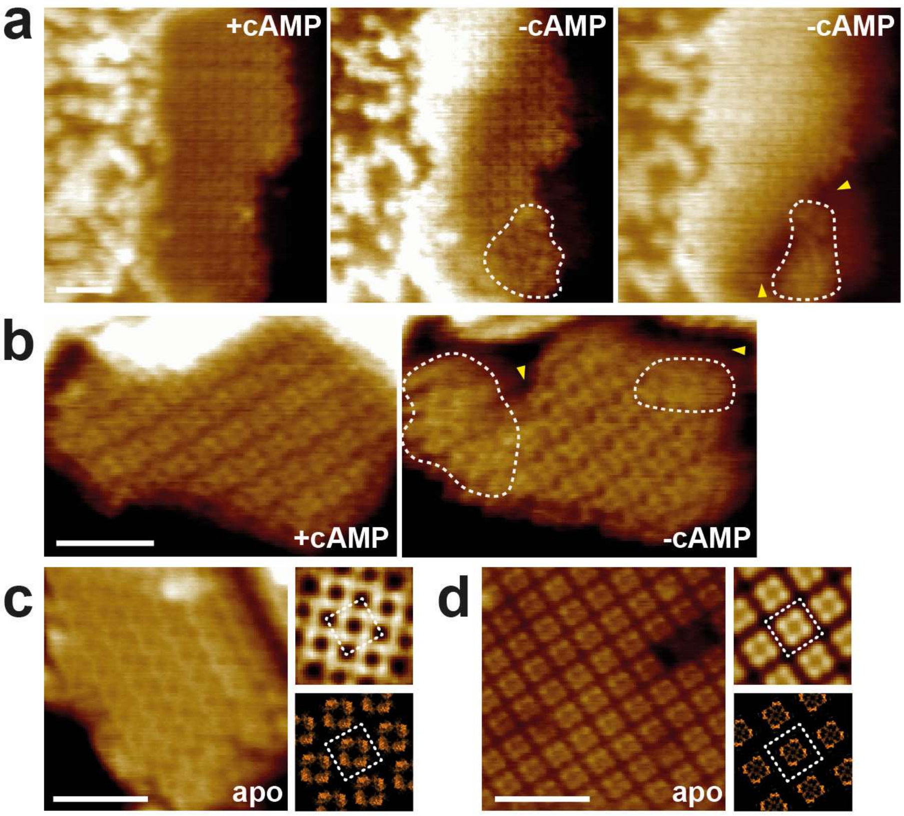

Supplementary Figure 3. Crystal packing changes upon cAMP removal. (a) and **(b)** Examples of two 2D-crystal patches showing transitions from +cAMP (0.1mM) to –cAMP (apo; no cyclic nucleotide) conditions (scale bars: 30nm). Despite difficulties to remove cAMP completely from the HS-AFM fluid cell and from the channel, the same conformational changes were observed as when outcompeting cAMP with cGMP (compare with Fig. 3). The dashed outlines delineate the membrane areas in which the activated to resting state transition has taken place. Yellow arrowheads indicate membrane incisions separating the activated and resting states molecules. Apo SthK 2D-lattice exposing the intracellular **(c)** and extracellular **(d)** faces (scale bars: 25nm). Top insets represent correlation averages and bottom insets display the packing models generated using the SthK apo cryo-EM structure (pdb 6CJQ), showing that the apo SthK structure viewed from both sides fits well the AFM topographs. The white dotted square outlines the same tetramer.

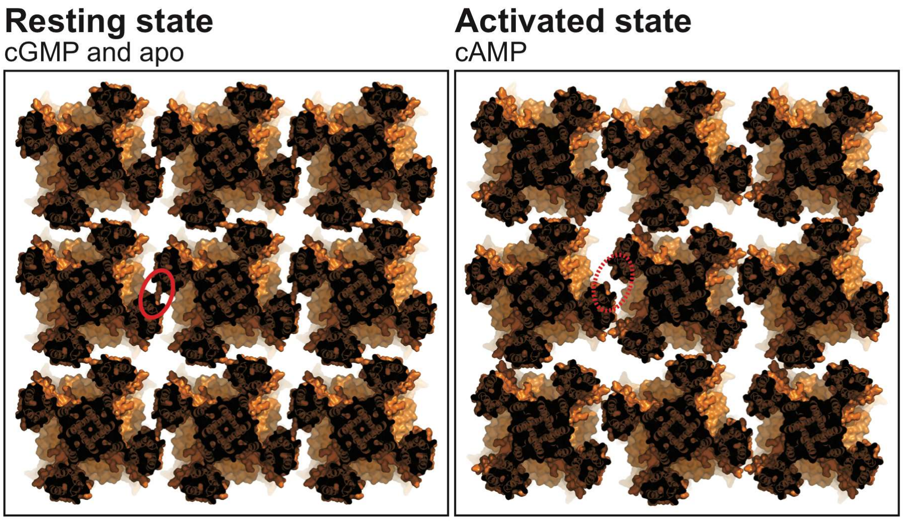

Supplementary Figure 4. Models of SthK packing arrangements in the resting (cGMP and apo) and the activated (cAMP) states. Shown is a slice through the crystal about half way through the membrane from the extracellular face. Both structural models were built by docking the resting state cryo-EM structure (PDB 6CJQ) into the correlation-averaged HS-AFM topographies. Given that the CNBDs undergo conformational changes in the activated state, we used the extracellular face HS-AFM topography, characterized by the small protrusions of the turrets between the S5 and pore helix that are most likely not undergoing any conformational rearrangements, to generate the activated state packing model (see Fig. 2b,d in the main manuscript). The resting state SthK channels form in-membrane 2D-crystal contacts with their VSDs, more specifically by elements in and around S3 (red solid outline, left). This protein-protein interaction is broken upon channel activation (red dashed outline, right).

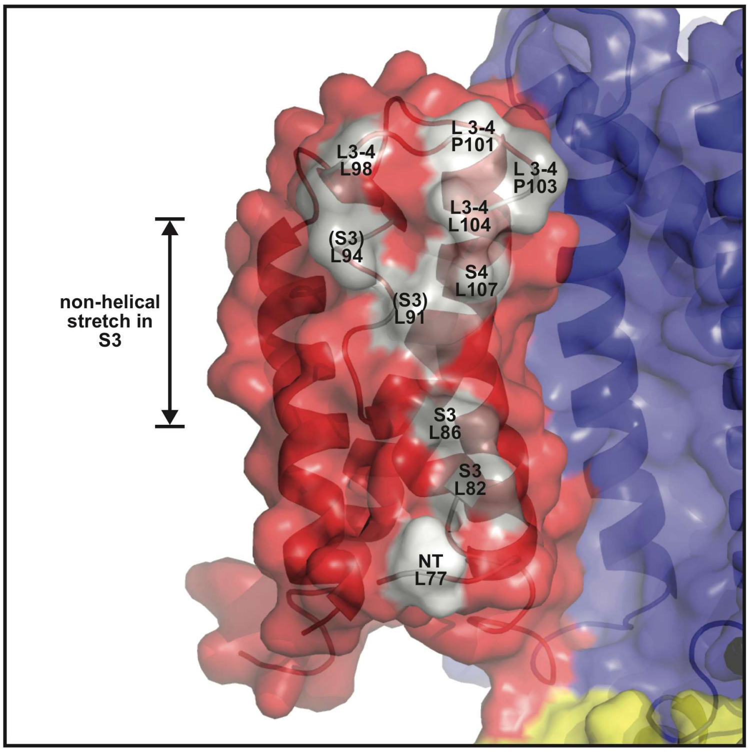

Supplementary Figure 5. Detailed view of the resting-state structure (PDB 6CJQ) VSD interface that makes protein-protein contacts in the resting-state packing. These protein-protein contacts are undone in the activated state. The key residues involved are labeled and colored in white and grey according to how far they protrude into the protein-protein contact region, respectively. In the N-terminus: Leu 77. In S3: Leu 82, Leu 86, Leu 91 and Leu 94. In the loop connection S3 and S4: Leu 98, Pro 101 Pro 103 and Leu 104. In S4: Leu 107. Note that the residues involved in the resting-state protein-protein contact are part of or surround the non-helical stretch from A88, L89, P90, L91, D92, L93, L94 to V95 in S3.
