# Supplementary material for: An iris diaphragm mechanism to gate a cyclic nucleotide-gated ion channel
Source: Nat Commun. 2018 Sep 28;9:3978. doi: 10.1038/s41467-018-06414-8 (PMC6162275; doi:10.1038/s41467-018-06414-8)
Supplement: Supplementary file 3 — Description of Additional Supplementary Files [file 41467_2018_6414_MOESM3_ESM.pdf]

Description of Additional Supplementary Files

File Name: Supplementary Movie 1
Description: **HS-AFM movie of SthK in a 2D-crystal during transition from the activated state (cAMP) to the resting state (cGMP)**. The HS-AFM fluid cell initially contained buffer with 0.1mM cAMP that was subsequently supplemented with 7mM cGMP. Left: Frames acquired at 1 frame / second. Right: 3-second time moving average. Image size: 200x175nm.

File Name: Supplementary Movie 2
Description: **HS-AFM movie of SthK in a 2D-crystal during transition from the activated state (cAMP) to the resting state (cGMP)**. The HS-AFM fluid cell initially contained buffer with 0.1mM cAMP that was subsequently supplemented with 7mM cGMP. During the experiment, parts of the membrane in which the molecules underwent the conformational transition to the resting state (cGMP) break off from membrane areas housing activated state (cAMP) SthK. Subsequently, all transit to the resting state (cGMP). Left: Frames acquired at 1 frame / second. Right: 3-second time moving average. Image size: 160x170nm.

File Name: Supplementary Movie 3
Description: **HS-AFM movie of SthK in a 2D-crystal during reversible transition from the activated state (cAMP) to the resting state (cGMP) and back to the activated state (cAMP)**. The HS-AFM fluid cell initially contained buffer with 0.1mM cAMP that was subsequently supplemented with 7mM cGMP. Following a constant pressure and constant flow pump was used to exchange the buffer again to excess 3.0mM cAMP and to dilute cGMP out. Left: Frames acquired at 1 frame / second. Right: 3-second time moving average. Image size: 140x165nm.
